# Post-Forming Mechanical Properties of a Polymer Sheet Processed by Incremental Sheet Forming: Insights into Effects of Plastic Strain, and Orientation and Size of Specimen

**DOI:** 10.3390/polym12091870

**Published:** 2020-08-20

**Authors:** Hongyu Wei, Ghulam Hussain, Behzad Heidarshenas, Mohammed Alkahtani

**Affiliations:** 1 College of Mechanical & Electrical Engineering, Nanjing University of Aeronautics & Astronautics, Nanjing 210016, China; behzadheidarshenas@nuaa.edu.cn; 2 Ghulam Ishaq Khan Institute of Engineering Sciences and Technology, Topi 23640, Pakistan; 3 Industrial Engineering Department, College of Engineering, King Saud University, Riyadh 11421, Saudi Arabia; moalkahtani@ksu.edu.sa; 4 Raytheon Chair for Systems Engineering (RCSE Chair), Advanced Manufacturing Institute, King Saud University, Riyadh 11421, Saudi Arabia

**Keywords:** incremental sheet forming, plastic strain, polymer, crystallinity, crystallite, micro-voids

## Abstract

The innovative Incremental Sheet Forming (ISF) process affects the post-forming properties of thermoplastic polymers. However, the effects of degree of plastic strain, and the orientation and size of specimen on the mechanical properties are still unknown. In the present study, therefore, the ISF process is performed on a polymer sheet by varying the plastic strain ranging from 6% to 108%. The corresponding effects on the properties and associated polymer structure are quantified by conducting a variety of mechanical and structural tests. The results reveal that the post-ISF tensile properties like yield stress, ultimate stress, drawing stress, elastic modulus and elongation decrease from 26.6 to 10 MPa, 30.5 to 15.4 MPa, 18.9 to 9.9 MPa, 916 to 300 MPa and 1107% to 457%, respectively, as the strain increases in the investigated range. The value of post-ISF relaxation properties, contrary to the tensile properties, increases with increasing strain up to 62%. Particularly, reductions in stress, strain and modulus increase from 41% to 202%, 37% to 51%, and 41% to 202%. As regard the orientation effect, the sheet in the feed direction shows greater strength than the transverse direction (up to 142% in yield stress and 72% in ultimate stress). Moreover, the smaller sample offers greater strength than the larger one (up to 158% in yield stress and 109% in ultimate stress). The analysis of the post-ISF tensile properties and structural results lead us to conclude that the drop in the tensile properties due to increasing strain occurs due to corresponding increase in the voids area fraction (1.25% to 31%) and a reduction in the crystallinity (38% to 31%).

## 1. Introduction

The trend of mass customization and rapid manufacturing is consistently rising in the market. On the other hand, the traditional processes mainly relying on the shape dependent tooling cannot meet these compelling demands. Therefore, new processes, characterized by flexibility, and capable of producing parts without using the dedicated tooling, need to be invented and deployed in the industry. In the 1990s, based upon the idea proposed by Leszak [1], Kitazawa et al. [2] invented die-less Incremental Sheet Forming (ISF) to produce sheet components. Being a die-less process, it reduces the lead time and eliminates the die/punch cost thereby enabling product delivery with short production cycles as compared with the traditional press forming operations. 

Although its invention was initially meant to shape the metallic materials [3,4], recent reports on the successful fabrication of thermoplastic components have unfolded a new research domain in the ISF process [5,6]. In fact, traditional processing of polymers relies on the shape dependent dies and heating-cooling cycles [7,8,9]. This does not only increase the product cost but also adversely affects the environment in terms of carbon emissions. On the other hand, ISF substantially reduces the tooling cost and minimizes the environmental burden by enabling cold processing [10,11]. Until now, process mechanics pertaining to polymers forming has been a major research focus of the researchers in this new domain. Silva et al. [12], in this regard, reported that yielding in polymers is controlled by pressure-dependent criterion. Marques et al. [13] found that an increase in stress triaxiality promotes damage and fracture in the polymers. Medina-Sanchez et al. [14] proposed a model to predict the forming force. Martins et al. [15] identified wrinkling and fracture as two failure modes in the ISF of thermoplastics. By conducting tests on two polymers, Hussain et al. [16] further explained that an increase in the softening index (i.e., the ratio of temperature and melting point) mitigates the fracture failure at the cost of an aggravation in the wrinkling failure. Many researchers have claimed that selection of the process parameters also affects the failure, especially the large-sized tool, greater step size, high spindle rotation and thinner sheet, promote the fracture failure [17,18,19]. 

The plastic forming processes affect the post-forming properties of the thermoplastics and thus, influence the service performance of the formed components [20]. The early investigation on this topic in ISF was undertaken by Devarpanah et al. [21]. They, keeping the strain to a fixed value, reported that the mechanical deformation causes a reduction in the tensile yield strength and tensile modulus, while an increase in the tensile ductility and ultimate tensile strength. Further, the formed material experiences more relaxation than the unformed one when subjected to a relaxation test. As regard the correlation between the process parameters and post-forming properties, the nature and degree of their effects are found to be material dependent [18]. From the structural analyses, they revealed that the deformation reorients the molecular chains, and further, voids are formed whose density increases with increasing of the step size, thereby causing a loss of post-forming yield strength [18,21]. Lozano-Sánchez [22] further studied the orientation of molecular chains in the formed polymers and clarified that the chains on the inner surface are preferentially aligned in the horizontal feed direction, reasoning to tool’s shearing action, while those on the outer surface are pulled downwards representing the dominant effect of sheet stretching. In another work, they proposed to reinforce polymers with CNTs (Carbon Nano Tubes) to minimize voids density and enhance thermal stability in the formed polymers [23]. Further, the CNTs proportion inversely affects the voids density. Boric et al. [24] introduced the idea of plasticization of polymers by adding foreign particles. They added clay Cloisite 93A in the polyamide and concluded that its addition from 1% to 3% increases the elongation from 44% to 49%, which in turn enhances the ISF formability. 

The post-forming mechanical properties play a vital role in the product design. Therefore, comprehensive knowledge on this subject is mandatory for realizing a robust design of a given product. Although, as discussed in the last paragraph, this subject has received attention in ISF of the polymers, the reported studies are fundamental and further work is required to comprehend the knowledge. The deformation effects on the post-ISF tensile properties have been undoubtedly investigated [18,21,23]; however, the strain level was kept constant throughout the investigations. In fact, according to the literature published on traditional cold forming of polymers, the degree of plastic strain substantially impacts the chain orientation and thus, the post-forming mechanical properties of polymers [20]. For an example, as presented in a review paper by Bigg [25], an increase in the draw ratio (i.e., strain) leads to an increase in the tensile modulus of various polymers. Rjabi et al. [26] reported similar results in deep drawing of thermoplastics. These findings on traditional forming of polymers follow that the strain degree may also affect the post-forming properties of the ISF polymers. Therefore, systematic investigations are required to ascertain this point with an objective to generate useful information for designing a product to be produced through the ISF process. 

As discussed above, the ISF process affects the chain orientation and further the orientations on the inner and outer surfaces are different [21,22] thereby signifying inducement of directional properties in a formed polymer component. Therefore, the dependence of resulting properties of a formed polymer on the direction or orientation of a specimen (say tensile specimen), with respect to the tool travel, should be investigated. 

Qdom and Adam [27] have reported that the size of a tensile specimen influences the post-forming strength in a way that the strength increases with decreasing the sample size reasoning to the fact that a large sample suffers more defects than a small one. How the specimen size affects the post-ISF properties of a polymer requires investigation, in order to examine if this finding applies to ISF parts and thus, to comprehend information for material characterization and product design.

The present study addresses these points intending to comprehend knowledge on ISF of the polymers to provide a guideline to the product designers and process users. Owing to excellent properties like high flexural strength, good fatigue and toughness, and resistance to chemical and moisture attack, polypropylene is a very suitable material for a variety of industrial applications (e.g., automotive parts, medical supplies and packaging). Moreover, being an inexpensive material, it is the second largest polymer after polyethylene produced in the industry. In the present study, therefore, the polypropylene sheet is employed as the experimental material. A range of plastic strain (6% to 108%) is applied on the sheet through the ISF process. The effect of plastic straining on the post-forming properties is characterized by performing a series of tension tests, and that on the structure, is analyzed by conducting Scanning Electron Microscopy (SEM), Differential Scanning Calorimetry (DSC), and X-Ray Diffraction (XRD) tests. To determine the effect of orientation and size of specimen, the samples are cut in two directions and in two sizes, respectively, which are then subjected to tension tests. The results show that the strain degree and other considered parameters, as expected, affect the post-forming properties and allow to detect an important factor that controls these properties. The reported results are useful for the design of the polymer components and helpful in determining the allowable strain from the perspective of the desired component strength.

The rest of the contents are divided into three sections. The material and experimental details are given in Section 2, and the experimental results, discussion and critical analysis are detailed in Section 3. Finally, the important conclusions are summarized in Section 4. 

## 2. Materials and Methods 

**Figure 1:**
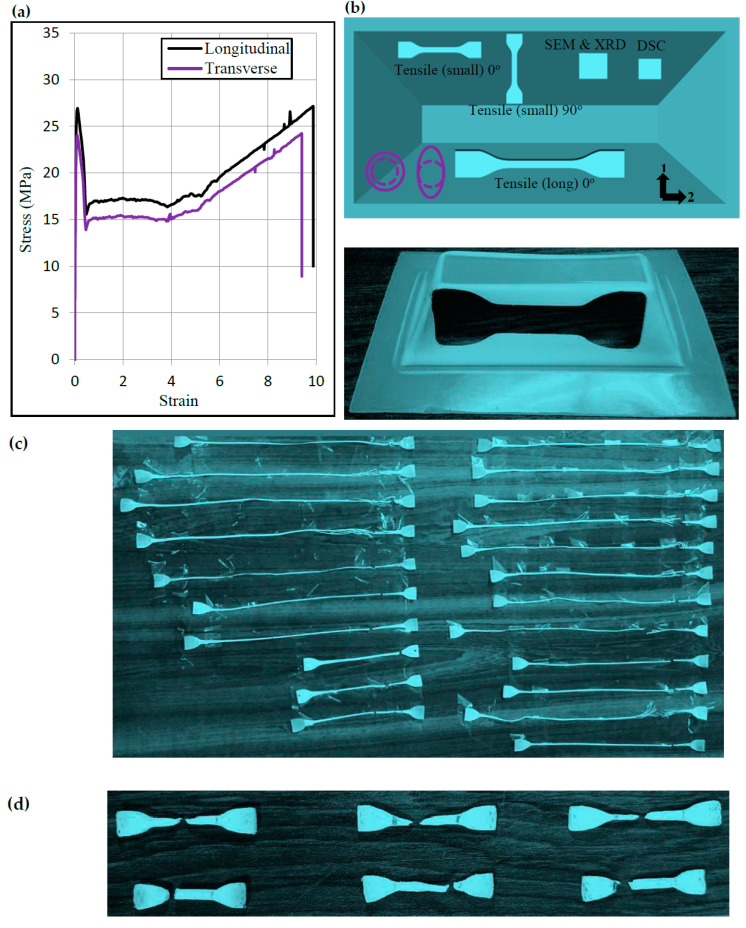
Experimental details: (**a**) Stress/strain curves of unformed sheet, (**b**) Test geometry with extraction plan of various specimens, (**c**) Representative long tensile specimens after tensile failure, and (**d**) Representative small tensile specimens after tensile failure. DSC: Differential Scanning Calorimetry.

The Polypropylene sheet was obtained from the supplier in the thickness of 2 mm. The sheet had comparable strength in the longitudinal (0°) and transverse (90°) directions, as noticeable from the stress-strain curves presented in Figure 1a. To perform ISF, the sheet was assembled in a rig with its longitudinal direction aligned with the longer side of the rig. The forming area of the sheet was 140 mm × 70 mm, which was deformed into a frustum of the pyramid with a slant height of at least 30 mm (Figure 1b). The forming was executed utilizing a steel rod with a hemispherical end as a forming tool, and a CNC milling machine as forming equipment (Dugard, Hove, UK). While forming, mineral oil was used as a lubricant to minimize friction at the tool/sheet interface. Since the forming parameters affect the degree of sheet straining [3], a series of tests with a variety of combinations as listed in Table 1 were executed. The range (s) of the parameters was set according to the literature recommendations, machine limits, and the author’s experience. For instance, the maximum rotational speed was set to 1500 rpm as further increase could cause galling of the sheet. The maximum tool diameter was 20 mm because there was a limitation on holding larger tools in the spindle of the machine. The high level of step size was 1 mm as excessively large steps can generate poor surfaces. Each of the tests were executed twice to provide statistical means to the results. 

The pyramid shape consists of flat surfaces and corners. As schematized in Figure 1b, a flat surface during ISF endures plane strain state, while a corner experiences a bi-axial strain state [3]. The present analysis was restricted to the plane-strain condition because this strain state is most common in the ISF components. To determine the strain that ISF induced in the flat surfaces of the pyramid, the thickness was measured with a vernier caliper with an accuracy of ±0.01 mm. The thickness was observed to vary from specimen to specimen thereby indicating that the thickness, and hence, strain depends on the forming conditions. Assuming plane strain state (i.e., ε_2_ = 0 and ε_1_ = −ε_3_) and ignoring bending effects, the effective plastic strain on the flat surface was determined by applying the below formula [12]:

(1) $$\varepsilon_{p}=\sqrt{\frac{2}{3}\left(\varepsilon_{1}^{2}+\varepsilon_{2}^{2}+\varepsilon_{3}^{2}\right)}=\frac{2}{\sqrt{3}}\varepsilon_{3}$$

where ɛ_p_ is the effective plastic strain applied on the sheet during ISF, and ɛ_3_ is the thickness strain, which was determined as below:

(2) $$\varepsilon_{3}=ln\frac{t}{t_{o}}$$

where, $t_{o}$ is the thickness of the unformed sheet and t is the thickness of the formed sheet.

The extraction plan of various types of samples is shown in Figure 1b. In order to determine the post-forming tensile properties of polymer sheet, tensile specimens conforming to the ASTM D638 (sub size) were milled from the formed pyramids. These samples were extracted along the longitudinal direction 0° (i.e., tool travel direction), as defined in Figure 1b. To find the effect of specimen size, tensile specimens with reduced size (3 times) were extracted in the longitudinal direction. To analyze the effect of orientation, tensile specimens were extracted in the transverse direction 90°. To examine the influence of strain level on post-forming relaxation properties, tensile specimens were cut in the longitudinal direction.

The thickness of the extracted tensile specimens was measured from the gauge section with a Vernier caliper with an accuracy of ±0.01 mm. The mean of this thickness was used to calculate stress in the tension test. The tension and relaxation tests were performed on the Instron Universal Testing Machine (Norwood, MS, USA). The extension speed during these tests was set as 15 mm/min. The specimens in tensile tests were stretched to fracture. The specimens in relaxation tests were stretched beyond the yield strain (≈50%) and were held at this strain for a time of 20 min to observe a drop in the stress. Upon unloading from the tensile machine, the length of the gauge section of the specimen was monitored and recorded at multiple intervals to calculate strain reduction over a time span of 20 min, as the specimens did not show any pronounced relaxation over an extended time. Representative tested specimens are shown in Figure 1c,d.

The XRD samples were cut in a size of 15 mm × 15 mm as depicted in Figure 1b. Philips Analytical PW 1830 XRD apparatus (Amsterdam, Netherlands) was employed to perform the XRD tests. The samples with a diffraction angle ranging from 15° to 80° were exposed to radiations (source: Cu-Kα) at the scanning speed of 0.05°/s and step time of 1 s. The tests were performed on the inner surface (one in contact with the forming tool) and in the two orientations by aligning the beam along 0° and 90° as explained elsewhere [22]. 

The DSC tests were conducted in a Mettler Toledo DSC 1 calorimeter in a nitrogen environment. To perform these tests, samples weighed to 8 mg were cut, as indicated in Figure 1b. These were then heated from −20 to 220 °C with a temperature ramp of 10 °C/min. The purpose of the DSC tests was to estimate the crystallinity of the polymer sheet. The enthalpies of melting and cold crystallization were determined and put into the following formula [18]:

Degree of crystallinity = (∆*H*_m_ − ∆*H*_cc_)/∆*H*°_m_ (3)

where ∆*H*_m_ is the melting enthalpy, ∆*H*_cc_ is the cold crystallization enthalpy, ∆*H*°_m_ is the melting enthalpy of 100% crystalline material, which is 207 J/g for the polypropylene sheet [28].

To study the microscopic changes that occurred in the sheet as a result of deformation, the samples were cleaned with acetone and were observed in a scanning electron microscope (SEM Model: MIRA3 TESCAN, Brno, Czechia). Prior to conducting SEM analysis, the polymeric samples were made conductive with copper plating.

## 3. Results and Discussion

### 3.1. Tensile Stress/Strain Behavior

**Figure 2:**
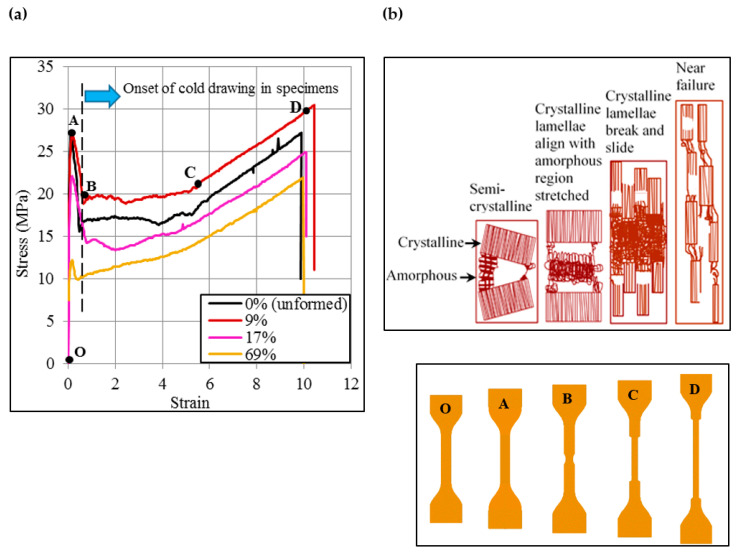
Stress/Strain behavior of unformed and formed sheets: (**a**) Stress/strain curves, and (**b**) Schematic of cold drawing and plastic deformation of molecular chains.

The tensile stress/strain behavior of the unformed polypropylene sheet and a representative set of formed sheets is presented in Figure 2a,b. There is a sudden drop in the stress after the onset of yielding thereby indicating the inception of necking, wherein fragmentation of crystalline lamellae into multiple segments occurs, regarded as cold drawing in plastic forming of thermoplastics. This has been shown in a schematic in Figure 2b (A–B). The neck later grows with the increasing tensile strain (B–C–D). In the early stage (B–C), the drawing in the unformed sheet (0% pre-tension plastic strain) and lightly strained sheets (say for pre-tension plastic strain ɛ_p_ of 9% and 17%) occurs either at almost constant stress or descending stress, representing the sliding of broken chain segments. However, once the sheet has endured a certain tensile strain (say up to point C), the stress sharply rises with the progression of the tension test, signifying enhancement in the load-carrying capacity of the sheet material due to stretching of the aligned chain segments. The stress in the severely plastically strained sheet (s) (say for ɛ_p_ of 69%), contrary to the lightly strained sheet (s), immediately begins to increase with the onset of cold drawing, pointing out that the molecular chains already had experienced tangible stretching and stiffening during ISF, prior to executing the tension test. This suggests that the chain fragmentation on the onset of drawing will reduce if the sheet endures the pre-tension strain. 

**Figure 3:**
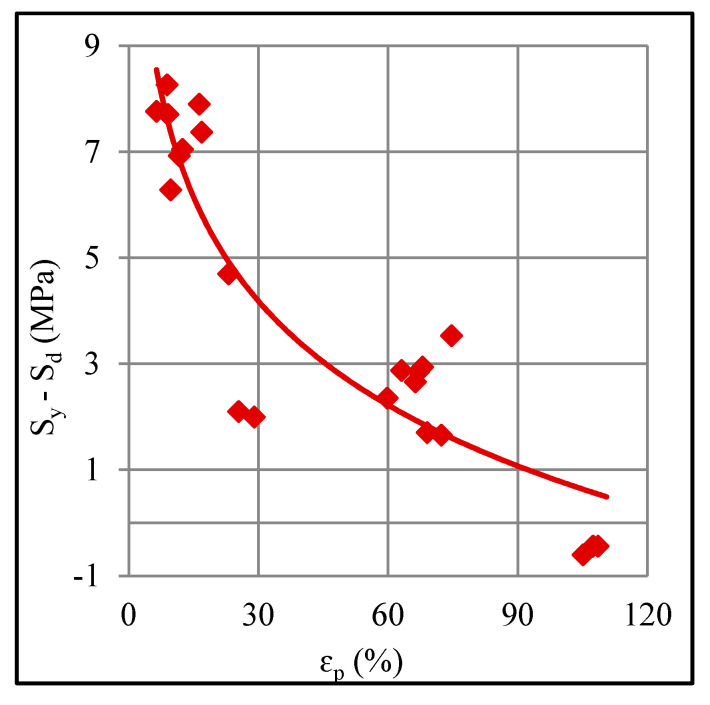
Correlation between the applied plastic strain and stress drop in tension test.

This argument is further supported by a fact that the stress difference *S*_y_–*S*_d_ reduces as the pre-tension strain increases (Figure 3) thereby showing that drawing stress *S*_d_ endures increasingly smaller losses than the yield stress *S*_y_, and thus, confirming that fragmentation on the onset of drawing correspondingly decreases. The stress–strain curves show a wavy pattern, which probably represents the localized breaking of molecular chains. This is more pronounced, especially from B–C, in the lightly strained sheets than that in the severely strained sheets.

### 3.2. Plastic Strain Effects on Mechanical Properties

**Figure 4:**
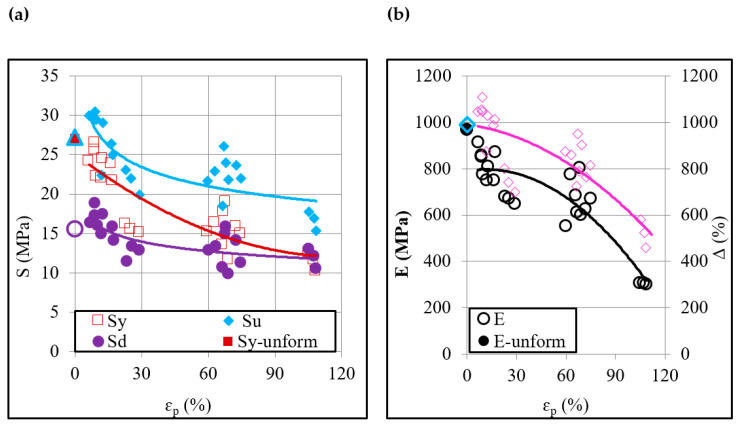
Correlations between the applied plastic strain and post-forming tensile properties: (**a**) Yield stress, ultimate stress and drawing stress, and (**b**) Elastic modulus and elongation.

Figure 4a,b depicts correlations between the plastic strain ɛ_p_, induced by ISF, and various post-forming tensile properties of the polypropylene sheet. As can be seen, the value of either of the stress measures namely yield stress *S*_y_ (26.6 to 10 MPa), ultimate stress *S*_u_ (30.5 to 15.4 MPa) and drawing stress *S*_d_ (18.9 to 9.9 MPa) decreases with increasing the plastic strain (6% to 108%). This means that the plastic deformation in ISF causes strain softening of the polypropylene sheet, contrary to strain hardening of metals [3,4]. This discrepancy can be reasoned to a fact that defects like dislocations density increases with increasing strain in the metals and raise the strength as a result [29]. The increase in plastic straining in polymers, on the contrary, generates defects like voids and crazes consequently leading to a drop in the strength, as will be shown later. The elastic modulus E (916 to 300 MPa) and tensile elongation ∆ (1107% to 457%) also reduce with the plastic strain. These results are in contradiction with the traditional forming of the polymers, especially extrusion and rolling, and may be attributed to an obvious difference in the state of the stress that is dominantly compressive in extrusion and rolling whereas tensile in ISF [15,20]. The elongation constitutes a similar relation with the plastic strain as presented by the stress and modulus thereby revealing that an ISF-formed sheet will endure a greater tensile strain if it endures greater tensile stress. This characteristic of ISF processing is interesting and holds contradiction with ISF of the metals whereby the plastic deformation makes dislocations piles and hinders the slip process consequently reducing elongation/ductility with an increase in the strain level [30].

**Figure 5:**
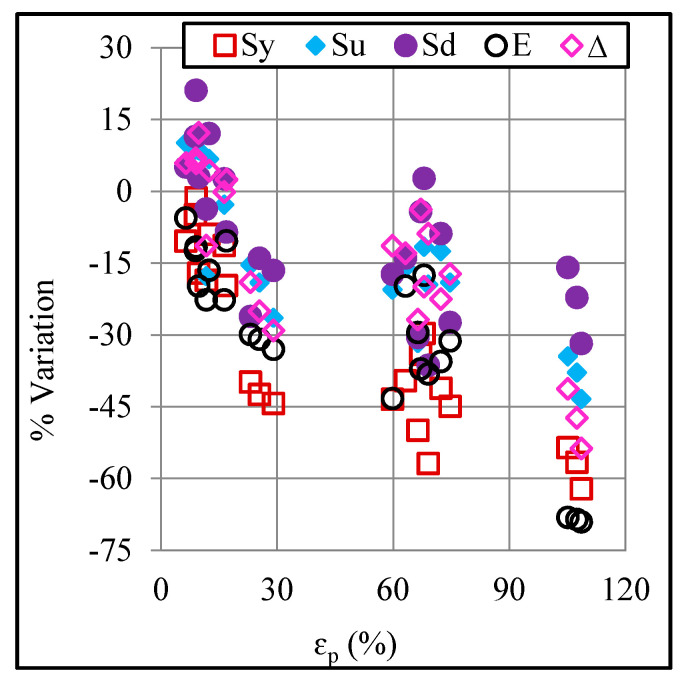
Variation in the tensile properties upon forming.

In order to analyze the effects of the ISF process on tensile properties of the Polypropylene sheet, the pre-forming and post-forming properties of the sheet were compared. As presented in Figure 5, the properties of the sheet endure a change upon forming. The yield stress ranges from −1.4% to −62%, the ultimate stress ranges from 12% to −43%, the drawing stress ranges from 21% to −36%, the elastic modulus ranges from −5.6% to −59%, and finally, the elongation ranges from 12% to −54%. These results reveal that both important design properties, namely yield stress and elastic modulus, received a drop over the entire range of applied strain and further, the respective drop increases with increasing the plastic strain. This can be attributed to increasing formation of defects (say voids, microcracks, and crazes) with increasing plastic strain, as will be shown in Section 3.4. The other tensile properties, however, show a varied dependence on the strain: ultimate stress, drawing stress and elongation endure a gain when the sheet is exposed to light strains (say ≤ 16%) but these quantities contrarily receive a loss in these properties when the sheet is exposed to severe strains. Devarpanah et al. [18] also noticed a drop in the yield stress and elastic modulus of PVC and polyamide sheets upon ISF. The present study has further elucidated that the drop in the mentioned properties increases as plastic strain in the sheet increases. Moreover, an increase in some of the tensile properties of the sheet can be also realized if strained lightly. 

**Figure 6:**
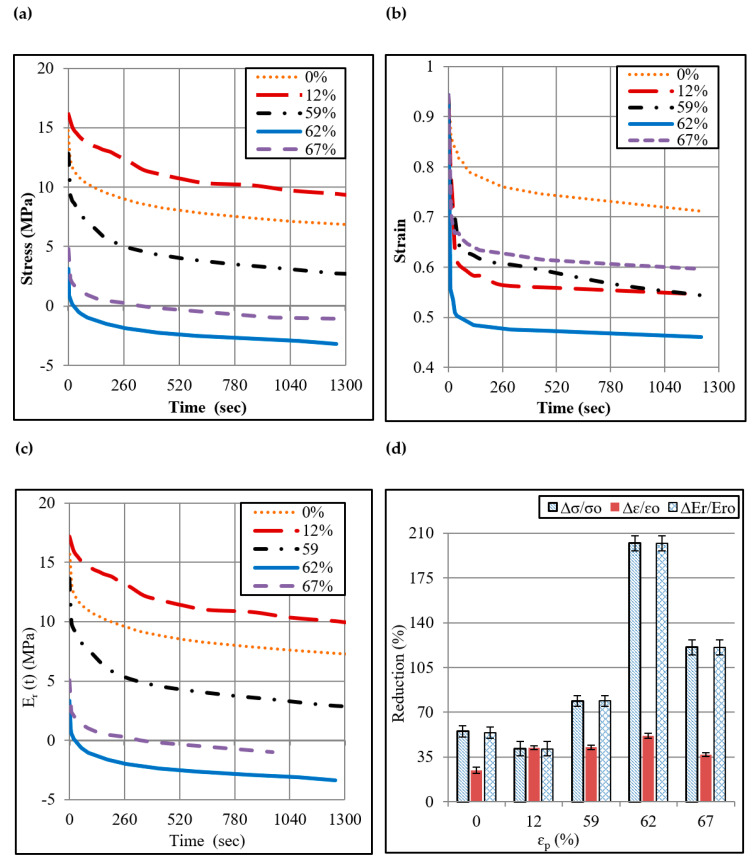
Relaxation behavior of Polypropylene sheet: (**a**) Stress relaxation curves, (**b**) Strain relaxation curves, (**c**) Relaxation modulus curves, (**d**) % Reductions in stress, strain and modulus.

The thermoplastics subjected to deformation are known to exhibit pronounced an-elastic recovery once the forming loads are removed. In order to determine how the plastic deformation in ISF affects the elastic recovery in polypropylene, relaxation tests were performed on the unformed and formed sheet (s) and the stress and strain reductions were recorded for a certain time period as mentioned in the previous section. As presented in Figure 6a–c, the magnitude of stress σ, strain ɛ, and relaxation modulus *E*_r_ (t) reduces with time. The reduction rate is higher in the beginning of the given time span, which according to the literature [20] happens because the elastic strain recovers rapidly. The rate reduces over time as the recovery of the plastic strain begins at a slower rate. The percent reductions in these quantities were determined as presented in Figure 6d. The stress reduction ∆σ/σ_o_ and strain reduction ∆ɛ/ɛ_o_ (σ_o_ and ɛ_o_ are the initial stress and strain at the start of the considered time span) in the formed sheet ranges from 41% to 202% and 37% to 51%, respectively, as the plastic strain ɛ_p_ increases. Likewise, reduction in the relaxation modulus ranges from 41% to 202%. As can be noticed from Figure 6d, the magnitude of these reductions with strain increases for a maximum of 62% as any further increase in the strain results in an opposite effect. In fact, two competing processes operate during deformation of polymers: (a) formation of defects, and (b) chains alignment and stranding. The defects generate the amorphous region and thus increase the elastic recovery, while chain alignment does the opposite [20]. Therefore, it seems that the relaxation behavior of polypropylene is inspired by defects for strain ≤ 62%, while it is controlled by chain alignment for strain > 62%. However, further work is required in future to thoroughly investigate the reason(s).

As compared with the unformed sheet, a formed sheet experiences greater relaxation in the test, thereby indicating that the molecular processes operate at a greater rate in the formed sheet than in the unformed sheet. 

As regard the significance of these results, the post-forming mechanical properties can act as a guideline for the product designers and process users. Moreover, the strain-properties relationships allow for determining the allowable strain for producing a component with desired properties. From the relaxation properties, one can understand the strain recovery behavior and thus can control the geometric accuracy of polypropylene components in specific and other plastic components in general. 

### 3.3. Size and Orientation Effects on Mechanical Properties

**Figure 7:**
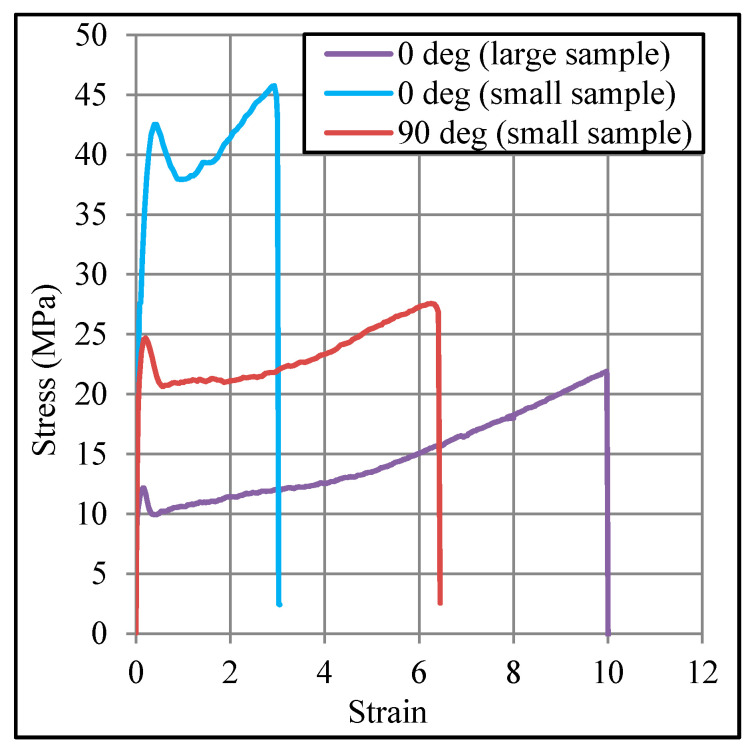
Stress/strain behavior comparison with respect to the size and orientation of tensile specimen: specimens extracted from sheet with 69% plastic strain applied in Incremental Sheet Forming (ISF).

In order to examine how the sample size and orientation affect the properties of the incrementally formed polypropylene, several tensile tests were performed by reducing the specimen size by three times. A set of representative stress/strain curves, as obtained from the tension tests, are presented in Figure 7. From the curves, it can be observed that the size of the specimen does not have any noticeable influence on the stress/strain behavior, but it affects the value of tensile properties. Similarly, the stress/strain behavior is insensitive to the orientation of the sample (0° and 90°); however, again, the magnitude of the properties is influenced.

**Figure 8:**
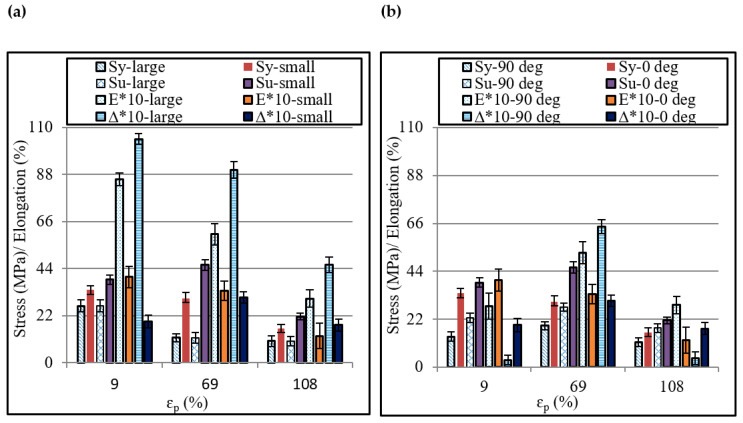
Effect of size and orientation of tensile specimen on the post-forming tensile properties: (**a**) Size effect, and (**b**) Orientation effect.

The size and orientation effects on various tensile properties are systematically shown in Figure 8. The small sample offers greater yield strength and tensile strength than the larger one (Figure 8a). Further, this difference in the strengths increases as the plastic strain increases. This ranges from 27% to 158% for the yield strength and 27% to 109% for the tensile strength in the given range of plastic strain (i.e., from 9% to 108%). The tensile elongation and elastic modulus contrarily experience a reduction as the sample size reduces. With increasing strain, the reduction in the two quantities is recorded to range from −61% to −81% and −44% to −58%, respectively. These variations, according to Qdom and Adams [27], occur due to the corresponding variation in the flaw’s density. Based on these results, it can be said that the sample size as expected affects the properties of the ISF-formed polymer. Further, it manifests that the size of an ISF component might also affect its service performance in terms of failure chances.

**Figure 9:**
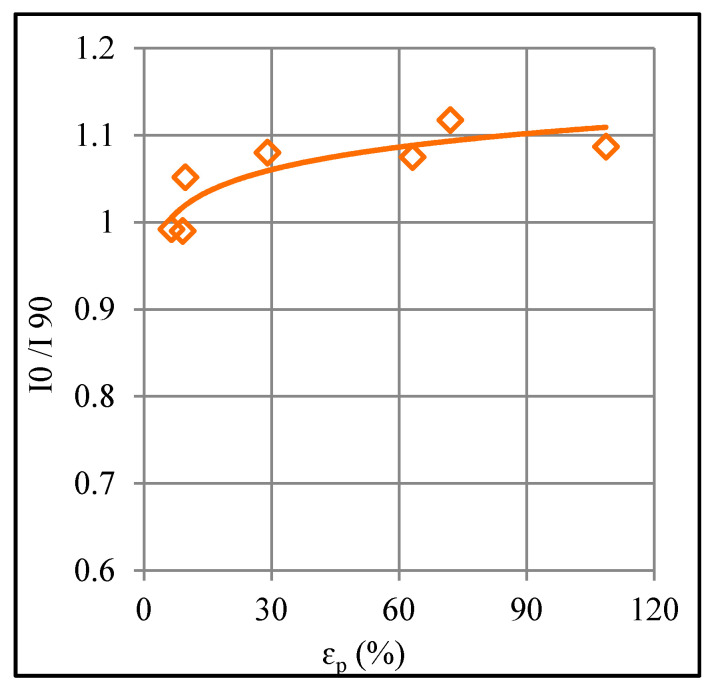
Plot between intensity ratio and plastic strain.

Regarding the orientation effect, the formed polymer shows greater strength in the longitudinal/feed direction (0°) than in the transverse direction (90°) (Figure 8b). The former direction offers 39% to 142% higher yield strength and 19% to 72% higher tensile strength than the latter direction. In order to find the reason for this finding, the orientation of chains in the formed polymers was analyzed. For this purpose, the XRD spectra were analyzed and the ratio of intensities in the two directions (i.e., *I*_0_/*I*_90_) was determined because this ratio can detect the chain orientation [22]. Figure 9 plots the ratio *I*_0_/*I*_90_ for a range of plastic strains. As can be observed, *I*_0_/*I*_90_ > 1 means the peaks intensity in the tool feed direction is greater than that in the transverse direction. Further, the forming tool dominantly aligned the chains in the feed direction thereby raising the load-carrying capacity and strength of polymer in this direction. As far as the elastic modulus and elongation are concerned, these do not show any consistent response to orientation change. From the design viewpoint, these results are important and suggest that the service loads should preferably be applied in the longitudinal direction to maximize the service life of a formed component. 

### 3.4. Structural Analysis

**Figure 10:**
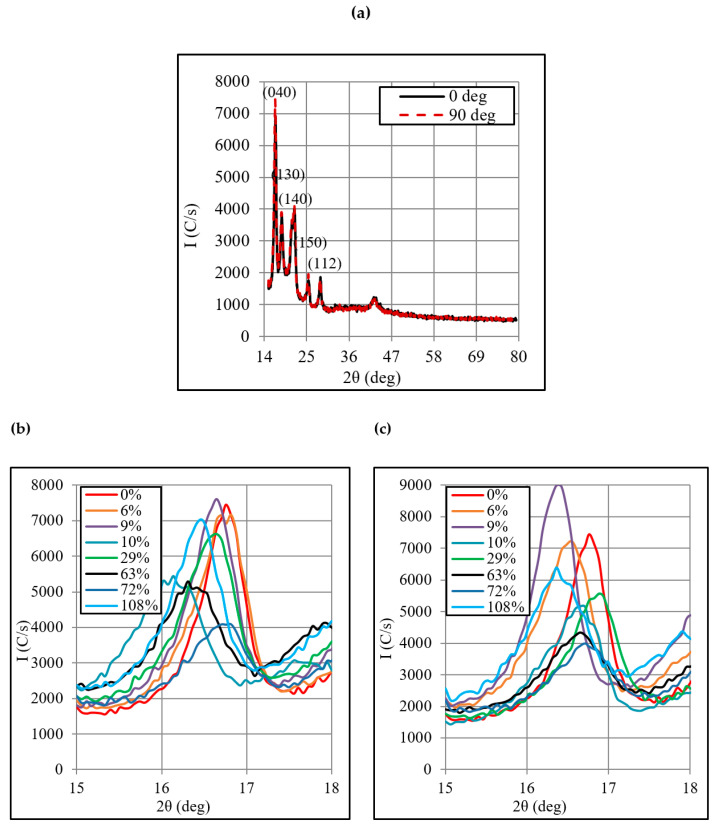
XRD patterns of unformed and formed sheets: (**a**) XRD pattern of the unformed sheet with 2θ range 15°−80°, (**b**) Enlarged view of XRD pattern of formed sheet in 0° direction with 2θ range 15°–18°, and (**c**) Enlarged view of XRD pattern of formed sheet in 90° direction with 2θ range 15°–18°.

Figure 10a–c presents XRD spectra for the unformed and formed Polypropylene sheets. The spectra of the unformed sheet (Figure 10a) shows that the diffraction peaks occurred at the diffraction angle 2θ of 16.7°, 18.3°, 21.5°, 25.1°, 28.3° and 41.6° indicating that crystalline regions were present in the material prior to forming. These regions are associated with the following hkl planes: (040), (130), (140), (150), and (112), respectively. The wide spans between every two consecutive peaks, specifically below 28.3°, represent amorphous regions thereby concluding that the polypropylene sheet was partially crystalline. According to the standard card, one peak associated with (110) plane should also occur at 14°, but it could not be observed in the present spectra because the sample was scanned in the 2θ range of 15° to 80°. Figure 10a shows that I_0_ ≈ I_90_ which means that the chains in the two directions were almost equally ordered before forming. However, upon forming, the intensity changes (i.e., *I*_0_ > *I*_90_) as observable from Figure 10b,c, thereby signifying chains alignment in the tool feed direction as discussed before. Moreover, the intensity changes after forming, thus qualitatively indicating that the forming process altered crystallinity of the polymer.

Besides the intensity, the diffraction angle 2θ corresponding to various peaks also undergoes a change, showing that the hkl planes were displaced due to deformation and affected the crystallite size, consequently. For further analysis, the crystallite size was, therefore, estimated in the two directions (i.e., 0° and 90°) applying the below Scherrer Equation [30]:

τ = *K*ʎ/βCosθ (4)

where *K* is a constant with the value of 0.9 rad, ʎ (1.54 Å) is the wavelength of radiations, θ is the diffraction angle, β is the inter-planar distance calculated using the below Bragg’s law [30]:

β = nʎ/Sinθ(5)

In this formula, n is the number of the interfering waves assumed to be 1. 

**Figure 11:**
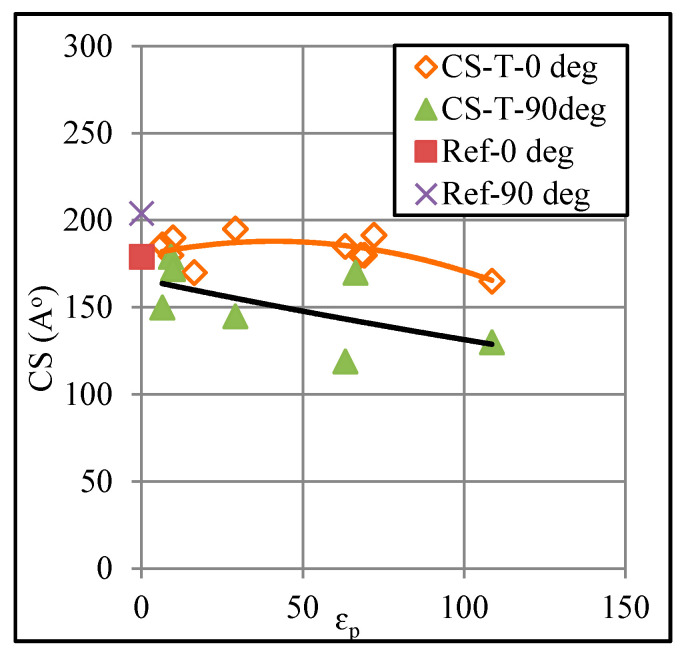
Correlation between crystallite size and plastic strain.

Figure 11 shows a relationship between the plastic strain and crystallite size. The crystallite size reduces from 179 to 165 Å as the plastic strain increases from 6% to 108%. Further, the crystallite size decreases in both the 0° and 90° directions. The maximum reduction in the crystallite size occurs against the largest strain, estimated to be about 19%. Coupling this strain-crystallite relation with the strain-strength relation presented in Figure 4, it can be said that the strength (both yield and ultimate) decrease as the crystallite size decreases. This result is contrary to the crystallite-strength correlation reported for the metallic materials [31,32] and thus points towards the possible effect of forming defects on reducing load-carrying capacity of the polymer to be discussed later. 

**Figure 12:**
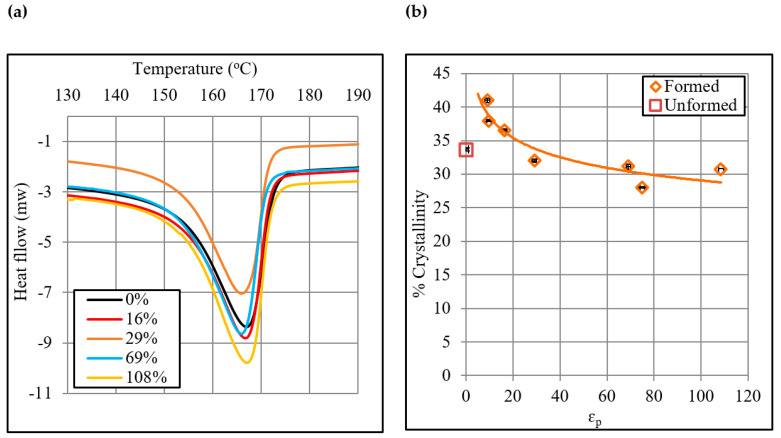
DSC results: (**a**) Thermograms, and (**b**) Correlation between % crystallinity and plastic strain.

Figure 12 shows the DSC results. The close-up view of thermo grams presented in Figure 12a depicts that the plastic strain affected the melting enthalpy of polypropylene, and it was estimated to range from 55 to 94 J/g. Similarly, the plastic strain affected the cold crystallization enthalpy as it was determined to range from 62 to 95 J/g. As a result of these enthalpy changes, the crystallinity of polymer changes. As presented in Figure 12b, the crystallinity decreases from 38% to 31% as the plastic strain increases from 9% to 108%. Thus, an overall reduction of 6.4% occurs in crystallinity due to the deformation of the sheet.

**Figure 13:**
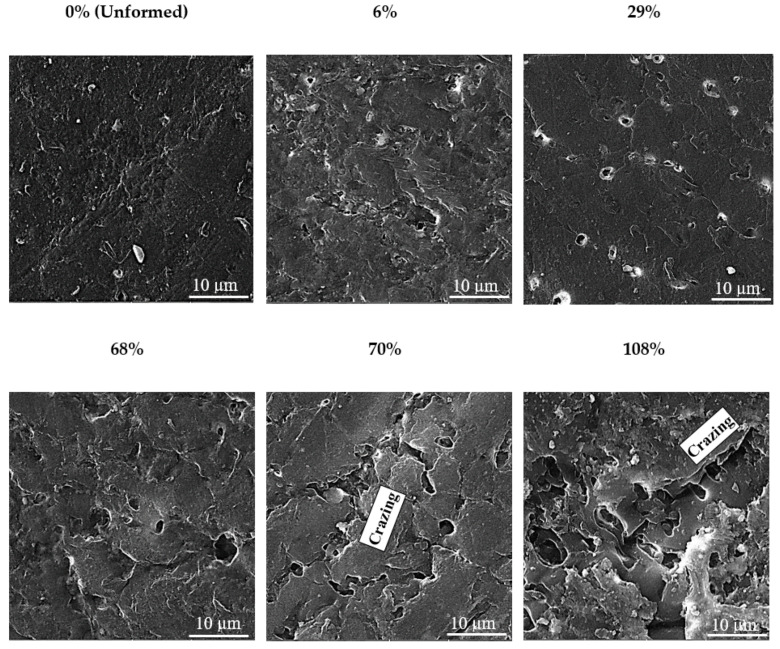
SEM images of the sheet surface at varying level of strain.

Figure 13 presents the SEM images of the unformed and formed sheets. As expected, the density and size of voids increase with increasing of the plastic strain. As estimated, the area fraction of voids (A/A_o_, where A and A_o_ are the area of voids and area of the sheet, respectively) increases from 1.25% to 31% as the strain increases from 6% to 108%. Moreover, crazing can also be observed at higher strains (say 69% and 108%). These defects consequently pose an adverse effect on the load-carrying capacity of the sheet as discussed before and shown in Figure 4. 

In summary, the crystallite size and crystallinity both decrease, while the voids area fraction increases with increasing plastic strain on the polypropylene sheet. Despite receiving refinement in the crystallite size at increased strain, the sheet experiences loss in the tensile yield strength and tensile modulus thus clearly following that the defects have a dominant effect over the mentioned post-forming properties. Moreover, coupling defects results with those of crystallinity, it is possible to say that loss of crystallinity with increasing strain occurs due to a corresponding increase in the defects density. 

## 4. Conclusions

The current study presented insights into the effects of plastic strain, and orientation and size of tensile specimen on the post-ISF tensile properties, relaxation properties and structure of the polypropylene sheet. These factors demonstrated a substantial impact on the mentioned properties. The post-ISF yield stress (26.6 to 10 MPa), ultimate stress (30.5 to 15.4 MPa) and drawing stress (18.9 to 9.9 MPa) decreased with increasing the plastic strain from 6% to 108%. Similarly, the elastic modulus (916 to 300 MPa) and elongation (1107% to 457%) also decreased with an increase in the plastic strain in the given range. These findings, according to the structural analyses, were reasoned to a corresponding increase in the voids area fraction (1.25% to 31%) and to a reduction in the crystallinity (38% to 31%). Further, the decrease in the considered properties with increasing strain, despite a corresponding increase in the crystallite size (179 to 165 Å), revealed that the voids area fraction was the dominant factor in controlling the post-ISF tensile properties of the polymer. The ISF process generally caused a loss of tensile properties of the polymer sheet. However, mild strains (i.e., ≤12%) proved conducive in realizing a certain gain in some of the properties especially ultimate stress (12%), drawing stress (5.6%) and elongation (12%). Therefore, one should follow this condition to enhance these properties if desired.

The ISF process generally raised the relaxation tendency of the polymer sheet. The relaxation properties like reductions in stress, strain and elastic modulus of the formed sheet increased from 41% to 202%, 37% to 51%, and 41% to 202%, respectively, as the plastic strain was increased up to 62%. These quantities, however, contrarily experienced a reduction on a further increase in the strain thereby showing the defects dominantly effect for strain ≥ 62%, and chain alignment dominantly effect for greater strains. However, this point needs more work in future for a thorough analysis.

The formed sheet showed greater strength in the feed direction than in the transverse direction (i.e., up to 142% in yield strength and up to 72% in ultimate strength), reasoning to a fact that the forming tool aligned the molecular chains in the feed direction. Moreover, a smaller tensile specimen, extracted from the formed sheet, showed greater strength than a larger one, i.e., up to 158% in yield stress and 109% in ultimate stress.

## Figures and Tables

**Table 1:** Tests performed and important results.

Test #	Φ (deg)	*f* (MPa)	ω (rpm)	*d* (mm)	*p* (mm)	*S*_y_ (MPa)	*S*_d_ (MPa)	*S*_u_ (MPa)	∆ (%)	*E* (MPa)
1	30	1000	0	6	1	21.62	14.25	24.94	1011	870
2	30	1000	1500	14	1	23.88	15.98	26.41	986	750
3	30	3500	0	14	1	25.62	17.36	29.55	1056	850
4	30	3500	1500	6	1	24.50	17.45	29	1030	810
5	30	3500	0	6	0.2	21.93	15	22.36	876	750
6	60	1000	1500	14	0.2	15.23	12.87	21.60	875	550
7	60	1000	0	14	1	17.77	14.91	26	950	610
8	60	1000	1500	6	1	15.85	14.20	23.76	765	625
9	60	3500	0	14	0.2	18.94	16	24.03	791	800
10	60	3500	1500	14	1	14.84	11.31	22	817	667
11	60	3500	0	6	1	13.48	10.82	18.56	723	684
12	60	1000	0	6	0.2	16.28	13.40	22.84	860	778
13	30	1000	0	14	0.2	26.57	18.87	30.48	1045	857
14	30	1000	1500	6	0.2	22.33	16.04	29.51	1108	778
15	30	3500	1500	14	0.2	24.13	16.37	29.92	1046	916
16	60	3500	1500	6	0.2	11.62	9.91	21.88	900	600
17	45	1000	0	6	0.2	15	13	20	700	650
18	45	1000	0	10	0.2	15.5	13.4	22	740	670
19	45	1000	0	14	0.2	16.2	11.5	23	800	680
20	70	1000	0	6	0.2	10.18	10.62	15.38	457	300
21	70	1000	0	10	0.2	11.68	12.12	16.88	520	305
22	70	1000	0	14	0.2	12.5	13.1	17.8	580	309

Note: Φ is the forming angle, *f* is the feed rate, ω represents the spindle rotation, *d* is the tool diameter, *p* stands for the step size, and *S*_y_, *S*_u_, ∆ and *E* are the tensile yield strength, tensile ultimate strength, tensile elongation and elastic modulus, respectively.

## List of Symbols

ISF	Incremental Sheet Forming
SEM	Scanning Electron Microscope
DSC	Differential Scanning Calorimetry
XRD	X-Ray Diffraction
*S*_y_	Tensile yield stress
*S*_u_	Tensile ultimate stress
*S*_d_	Tensile drawing stress
∆	Tensile elongation
*E*	Tensile elastic modulus
ɛ_p_	Plastic strain
*t*	Thickness of formed sheet
*t*_o_	Thickness of unformed sheet
*A*/*A*_o_	Area fraction of voids in the polymer sheet
*I*_o_	Intensity while X-ray beam aligned along feed direct
*I*_90_	Intensity while X-ray beam aligned along transverse direct
θ	Diffraction angle
